# Prediction of the Interface Shear Strength between Ultra-High-Performance Concrete and Normal Concrete Using Artificial Neural Networks

**DOI:** 10.3390/ma14195707

**Published:** 2021-09-30

**Authors:** Changqing Du, Xiaofan Liu, Yinying Liu, Teng Tong

**Affiliations:** 1 State Gird Jiangsu Electric Power Engineering Consulting Co., Ltd., Nanjing 210003, China; 220211347@seu.edu.cn (C.D.); liuxiaofanhd@163.com (X.L.); yayaliuying@163.com (Y.L.); 2 School of Civil Engineering, Southeast University, Nanjing 210018, China

**Keywords:** UHPC–NC, interface, bond strength, neutral networks

## Abstract

The bond strength between ultra-high-performance concrete (UHPC) and normal-strength concrete (NC) plays an important role in governing the composite specimens’ overall behaviors. Unfortunately, there are still no widely accepted formulas targeting UHPC–NC interfacial strength, either in their specifications or in research papers. To this end, this study constructs an experimental database, consisting of 563 and 338 specimens for splitting and slant shear tests, respectively. Moreover, an additional 35 specimens for “improved” slant shear tests were performed, which could circumvent concrete crushing and trigger interfacial debonding. Additionally, for the first time in our tests, the effect of casting sequence on UHPC–NC bond strength was identified. Based on the database, an artificial neural network (ANN) model is proposed with the following inputs: namely, the normal stress perpendicular to the interface, the interface roughness, and the compressive strengths of the UHPC and NC materials. Based on the ANN analyses, the explicit expression of UHPC–NC bond strength is proposed, which significantly lowers the prediction error. To be fully compatible with the specifications, the conventional shear-friction formula is modified. By splitting the total force into adhesion and friction forces, the modified formula additionally takes the casting sequence into account. Although sacrificing accuracy to some extent compared to the ANN model, the modified formula relies on a solid physical basis and its accuracy is enhanced significantly compared to the existing formulas in specifications or research papers.

## 1. Introduction

Recent decades have witnessed the emergence of ultra-high-performance concrete (UHPC) as a promising cementitious material in buildings [1], bridges [2], and tunnels [3], etc., due to its excellent tensile and compressive strength [4], agreed post-cracking behaviors [5], and enhanced durability [6]. Extensive application of UHPC materials to fully replace normal-strength concrete (NC) would render the cost prohibitively high [7]. Therefore, composite UHPC–NC has aroused the attention of engineers, in terms of, but not limited to, retrofitting existing structures [8], enhancing durability [9], and accelerating construction speed [10].

These UHPC–NC composite members, especially those without any rebars crossing their interface, would possibly face the risk of debonding when subject to larger external loading [11]. Interfacial debonding is a brittle failure mode and leads to premature failure of specimens far below their ultimate strengths [12,13,14,15]. Accurate prediction of interfacial strength between UHPC and NC is critical to estimate the composite specimen’s strength.

It is recognized that many design specifications, namely CEB-FIP [16], Eurocode 2 [17] and AASHTO LRFD [18] present similar formulas for predicting bond strength between new and old NC. What is more, numerous tests have been performed by researchers, targeting the bond strength between concretes [19,20]. Compared to older studies of bond strength between concretes, there is still no consensus on interfacial behaviors between UHPC and NC.

**Figure 1:**
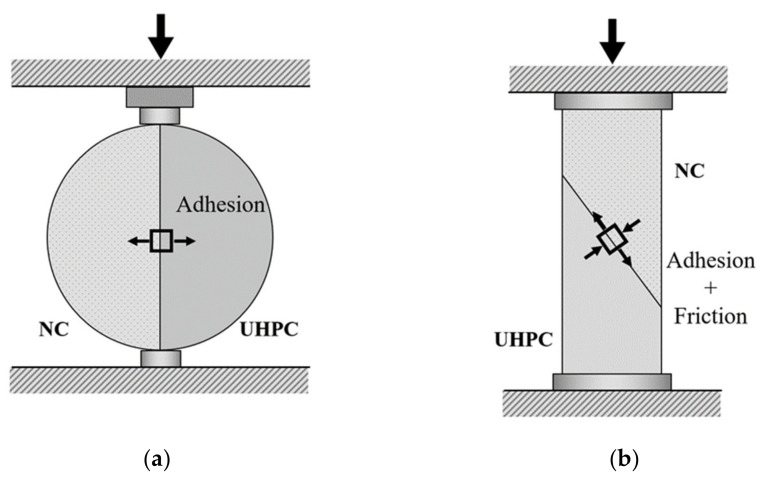
Testing methods for UHPC–NC bond strength. (**a**) Splitting test; (**b**) slant shear test.

The most commonly adopted testing methods to quantify the interfacial bond strength between UHPC and NC materials are the splitting tensile test and the slant shear test [21,22]. The splitting tensile test evaluates the adhesion between UHPC and NC materials subject to direct tension, without any contribution from friction force, through compression of a cylinder [23], as shown in Figure 1a. Comparatively, the slant shear test allows the combination of adhesion and friction forces, which renders a uniform distribution of shear-induced stress along the interface [24]; see Figure 1b.

Hundreds of specimens have already been loaded to failure, subject to the splitting test or slant shear test. To name a few, the effect of surface texture on mechanical bond characteristics was discussed by Tayeh et al. [25] and Harris et al. [26]. The influence of material strength was also tested by Zhang et al. [27] and Aaleti and Sritharan [28]. Some other experimental research has been conducted to analyze other parameters; Farzad et al. [29] evaluated bond strength with different interface moist conditions by splitting test. Carbonell Munoz et al. [22] investigated the development of the interface bond strength over time. However, systematic analyses of these important experimental data are still lacking. Although numerous factors were considered in previous studies, more experiments are still indispensable to identify further potential influencing factors. For example, the authors could not find relevant experiments for the effects of casting sequence (UHPC on existing NC structures or vice versa) on UHPC–NC bond strength [22,28,30,31].

Although different values are continuously proposed for UHPC–NC bond strength, they aim at individual experimental results from limited specimens [32]. Moreover, the shear-friction formula used in mainstream specifications [16,17,18] aims at the conventional interface between NC materials, rather than the UHPC–NC interface. Therefore, an accurate prediction model accounting for various influencing factors is urgent. Recently, artificial neural network (ANN) models are of interest to researchers for modeling various complex systems. To name a few, Naderpour et al. [33] applied an ANN model to predict the compressive strength of environmentally friendly concrete. A similar ANN model was applied to evaluate the compressive strength of recycled concrete aggregates in Hammoudi et al. [34]. Moreover, an ANN model was adopted to estimate the bond capacity between rebar and concrete [35,36] and the complex behaviors of FRP [37], UHPC [38]. Particularly for estimating the complex bond strength between concrete and repair overlay, the ANN model works as a complement and an alternative to conventional approaches. Jahangir, Eidgahee [39], and Haddad and Haddad [40] evaluated the bond strength between an FRP sheet and concrete substrate with an ANN model. Sadowski, Hoła [41] and Sadowski et al. [42] assessed pull-off adhesion between concrete layers cast at different times using an ANN method. Czarnecki et al. [43] presented the application of ANN for the non-destructive identification of pull-off adhesion values between a repair overlay with variable thickness and a substrate in concrete surface-repaired elements. Raab et al. [44] investigated the long-term behavior of the interlayer bond between asphalt pavements with an ANN method. The ANN model was also adopted by Sierra-Beltran et al. [45] to describe the bonding behavior of a bio-based mortar repair system for concrete. All these studies have proved that the ANN method can be used to predict interface shear bond properties between concrete and repair materials [46]. 

ANN models automatically adapt the data for training and can identify the complex relationships between input variables. ANN models are powerful enough to predict the bond strength between UHPC and NC materials, supposing that the main influence factors are properly selected. Although with wide applications, it is recognized that ANN models are implicit in nature, and behave like a “black box” for prediction, which is not suitable or convenient for engineering practices [47].

In this paper, the authors construct an extensive database regarding interfacial performance between UHPC and NC materials, consisting of 563 splitting specimens and 338 slant shear specimens (Section 2). Moreover, another 35 specimens were prepared for “improved” slant shear tests and the casting sequence was identified for the first time (Section 2). An ANN model was proposed which greatly improved prediction accuracy compared to existing formulas in either specifications or research papers (Section 3). Explicit expression of the bond strength is derived based on the ANN model, with several main factors being identified (Section 3). Finally, the conventional shear-friction formula is modified for the UHPC–NC bond strength, which has a solid physical basis and can be adopted for engineering purposes (Section 4).

## 2. Experiment and Database Construction

### 2.1. “Improved” Slant Shear Test

**Figure 2:**
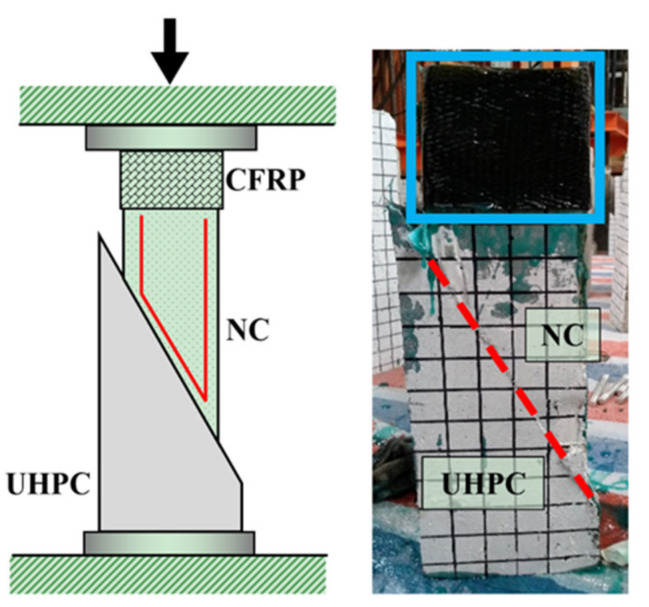
“Improved” slant shear test.

Realizing that tests regarding UHPC–NC bond strength were insufficient, in this study, we performed further “improved” slant shear tests containing thirty-five specimens. Figure 2 shows the specimens in detail. In particular, the following improvements were considered for these specimens:

(i) The NC component was wrapped with CFRP materials close to the loading surface;
(ii) The NC component was embedded with U-shaped rebars close to the interface; and
(iii) The top and bottom components had different cross-sections.

These three strategies render our specimens different from other conventional specimens in slant shear tests [28,29]—the reason lying in the fact that “local crushing” occurs inside the NC component close to the interface or close to the loading surface in these conventional specimens, rather than interfacial debonding [48], which would impair accurate prediction of real UHPC–NC bond strengths. With these “improved” specimens, debonding was found during the test and concrete crushing was circumvented, as expected.

The mean cubic compressive strength of NC, obtained from eighteen 150 mm × 150 mm × 150 mm cubic samples, was 54.6 MPa, which was equivalent to a cylindrical strength of 34.9 MPa. The UHPC powder was provided by Sobute New Materials Co., Ltd. (Nanjing, China); one cubic meter UHPC contained 2095 kg UHPC power, 156 kg steel fiber, 22.10 superplasticizer and 182.4 kg water. The mean cylindrical strength of the UHPC was measured at around 102.5 MPa.

In our test, the specimens were all 400 mm in height and took three different angles between UHPC and NC components into account, namely, β = 50°, 60°, and 70°. The bigger part had a cross-section of 150 mm × 100 mm while the smaller part was only 100 mm × 100 mm. Furthermore, the following four different surface treatments were accounted for: (i) smooth surface without any special treatment (S); (ii) electric breaker (E); (iii) hand-scrubbed (HS); and (IV) hand-drilled (HD). Different from other experimental studies [28,49], a new factor, casting sequence, was considered for the first time. Table 1 summarizes the details for the thirty-five UHPC–NC slant shear specimens. Taking specimen “NU50S” for example, it shows that the NC component was cast first, followed by the UHPC component, whereas no special treatment was performed at their interface of β = 50°.

The peak normal stress σ and shear strength τ are therefore obtained as:

(1) $$\sigma =\frac{P_{\max}\times \cos\beta }{A_{0}/\cos\beta }\;\mathrm{and}\;\tau =\frac{P_{\max}\times \sin\beta }{A_{0}/\cos\beta }$$

where $P_{\max}$ is the peak load of the specimen, and $A_{0}$ is the projected area perpendicular to the external load (*A*_0_ = 100 mm × 100 mm).

The tested results of these specimens are given in Table 2. As expected, the specimens without any surface treatments exhibited the lowest strength, e.g., $\tau_{\max}$ = 7. 33 MPa for specimen NU70S, 9.88 MPa for specimen NU70E, 14.03 MPa for specimen NU70HS, and 13.23 MPa for specimen NU70HD. Another interesting finding is that the casting sequences indeed affected the interfacial strength; the shear strength was $\tau_{\max}$ = 9.88 MPa and 14.58 MPa for specimens NU70E and UN70E, respectively. Similar trends were also observed in groups NU60E and UN60E, and groups NU50E and UN50E. It was first identified that specimens with NC cast first, followed by UHPC, exhibited higher interfacial strength than their counterparts with the opposite casting sequence. This will be discussed in Section 4.1.

### 2.2. Database for UHPC–NC Splitting Test

In splitting tests, UHPC and NC are cast together to form a cylinder sample (see Figure 1a). A pair of compressive forces is applied along the interface. The splitting test is an agreed method for quantifying adhesion under direct tension (Mode-I), but with friction force [22]. Resorting to the elastic Timoshenko’s equation, Carmona [50] states the relationship between adhesion and applied load as:

(2) $$\tau_{c}=\frac{2P_{\max}}{\pi A_{0}}$$

where $\tau_{c}$ is the adhesion under direct tension, *P*_max_ is the maximum applied load, and $A_{0}$ is the area of the interface between these UHPC and NC semi-circles.

In this study, a total of 563 specimens for UHPC–NC splitting tests were collected from the literature from over the past few decades. The following potential influencing factors were considered in these tests: namely, surface roughness, freeze–thaw cycles, ages, material strengths, and environmental moisture, as shown in Table 3. Since this study focuses on UHPC–NC bond strength for specimens subject to normal environmental conditions, we merely take the surface roughness and material strengths into account.

**Figure 3:**
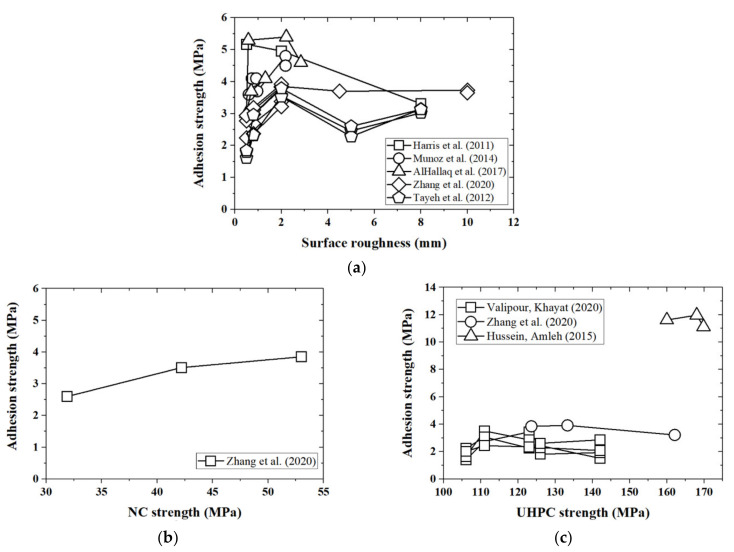
Relations between adhesion and different factors in the splitting test. (**a**) Adhesion and surface roughness; (**b**) adhesion and NC strength; (**c**) adhesion and UHPC strength.

The surface roughness R of the UHPC–NC interface is identified as the main factor influencing the extent of adhesion in the database. Zhang et al. [49] suggests the adoption of an average macrotexture depth to evaluate surface roughness. Figure 3a presents the trend between surface roughness and adhesion. It can be seen that adhesion increases with surface roughness, before R≥ 2.0 mm. When surface roughness is greater than 2.0 mm, its relationship to adhesion becomes complex; experimental results from Tayeh et al. [25] even show a decreasing trend when R≥ 2.0 mm (see Figure 3a). Similar phenomena are also observed in the NC–NC interface, reported by Júlio et al. [55]. Adhesion is mainly attributed to the chemical effect in the transition layer [56]. Compared to the as-cast surface (R≪ 2.0 mm), roughening treatments lead to an increase in cavities [57]. Positive ions inside these cavities improve the hydration process and also result in stronger chemical bonds around the interfacial transition zone [58]. It is also noteworthy that some global roughening treatments, such as sandblasting, wire-brushing, and power-washing, lead to a roughness R≈ 2.0 mm, whereas local treatment, such as drilling and grooving, lead to rougher surfaces with R≥ 2.0 mm. 

The effect of the compressive strength of the NC material $f_{\mathrm{NC}}$ on the UHPC–NC bond strength is only reported by Zhang et al. [49] (see Figure 3b). In their test, splitting tests on specimens were performed at $f_{\mathrm{NC}}$ = 31.9, 42.2, and 53.0 MPa, respectively. Their corresponding adhesions were measured as 2.60, 3.51, and 3.85 MPa, respectively. 

The effects of the strength of the UHPC material $f_{\mathrm{UHPC}}$ on UHPC–NC bond strength are reported by Hussein and Amleh [52], Valipour and Khayat [54], and Zhang et al. [49] (see Figure 3c), with different scales on the vertical axis. Adhesion apparently seems to be affected by the UHPC strength [29]. The curves show that adhesion first increases with UHPC strength, and then exhibits a decreasing trend. UHPC with higher strength is generally accompanied by a lower water–cement ratio [59], which could lead to a weaker interface [60,61]. 

Figure 3 shows that several governing factors may affect the UHPC–NC bond strength; however, their trends are very complicated and cannot be simply described with traditional methods in a quantitative way.

### 2.3. Database for UHPC–NC Slant Shear Tests

Specimens for the slant shear test allow the combination of compression and shear states at the interface (see Figure 1b) which mimic the stress state of real structures. The slant shear test is also deemed the standard method to evaluate the bond strength between repair materials. The normal stress σ and shear strength τ at the interface are evaluated in (1).

In total, 338 specimens were collected to form the database for the slant shear test, as shown in Table 4, with similar potential influencing factors being considered in Section 2.2. Here, we also do not consider the environmental conditions, but the surface roughness, material strengths, and normal stress level.

**Figure 4:**
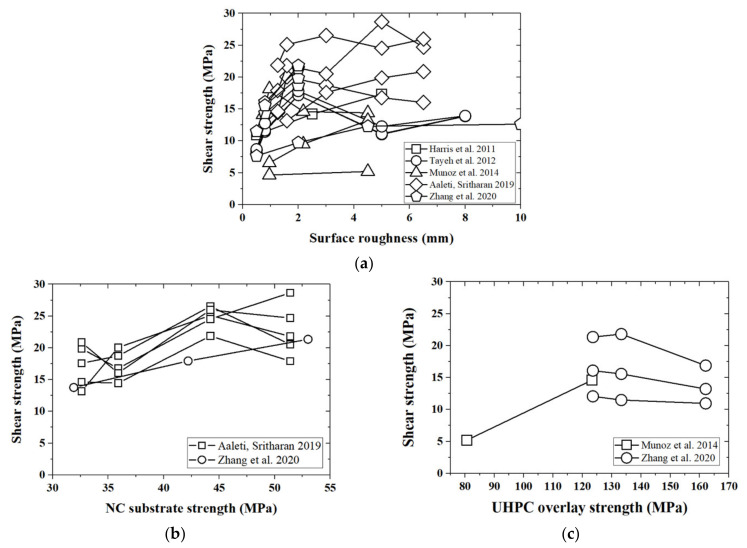
Relationships between shear strength and different factors in the slant shear test. (**a**) Shear strength and surface roughness; (**b**) shear strength and NC strength; (**c**) shear strength and UHPC strength.

Parametric studies for the slant shear test are also performed in this study. According to Coulomb’s law, surface roughness would have more apparent effects on UHPC–NC bond strength in the slant shear test [62]. Figure 3a clearly shows that increasing surface roughness with R≪ 2.0 mm positively affects the shear strength. However, the case for a surface roughness greater than 2.0 mm becomes more complicated; the trend observed in the slant shear test is similar to the phenomenon in the splitting test, as shown in Figure 4a. 

Figure 4b,c depicts the trends for shear strength with respect to the compressive strengths of NC and UHPC materials, respectively. Generally, shear strength increases with the rise of the compressive strength of the NC materials (see Figure 4b); however, its relationship with the compressive strength of the UHPC material is more complicated, without a clear or straightforward description. In a word, the collected database of the slant shear test could not identify the effects of the compressive strengths of UHPC and NC materials clearly and quantitatively.

## 3. ANN Modeling

### 3.1. ANN Training

**Figure 5:**
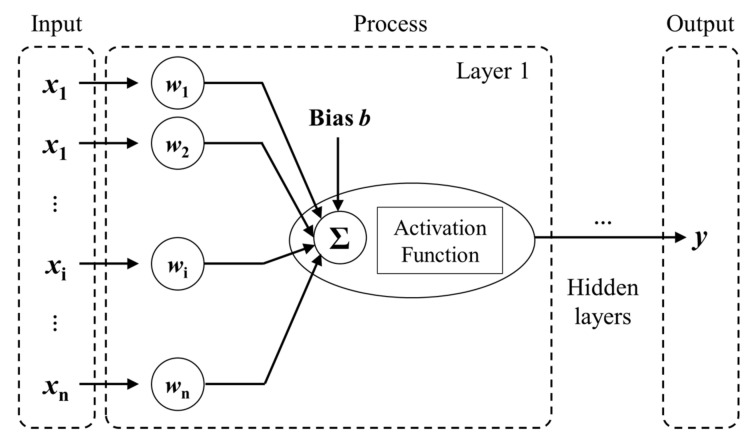
A sketch of the ANN model.

ANNs, which are also known as neural networks, are information processing systems inspired by the biological nervous system. ANNs are powerful at handling phenomena with complex influencing factors [47]. ANNs consist of artificial neurons, which serve as the base units. A simplified structure of the ANN model is illustrated in Figure 5, which consists of multiple inputs, and a nonlinear element for the single output.

Each input signal $x_{i}$ of the previous neural network layer is multiplied by the weight coefficient $w_{ij}$ at the j-th layer to produce weighted signals that are fed into the hidden neurons; the summation function is then applied to these weighted input signals. The activation function $f\left(x\right)$ is adopted to illustrate nonlinear behavior. This calculation process is repeated for each layer to produce the final output of this ANN model, which can be expressed as [47]:

(3) $$y=f\left(\overset{n}{\underset{i=1}{\sum }}\left(w_{ij}x_{i}+b\right)\right)$$

where b is the bias and can be defined as a type of connection weight with a constant nonzero value added to the summation of inputs and corresponding weights.

In this research, an ANN model was constructed for the splitting tensile and slant shear tests database presented in Section 2. According to research by Pham and Hadi [63], one hidden layer was selected to obtain an accurate and satisfactory result. A trial-and-error approach was applied to the ANN model in this paper, which takes 3 to 30 different hidden layer nodes for parametric training with a set of random initial weights. Based on the discussion in Section 2, four potential factors were selected as the input signals: interface roughness R, normal stress σ, NC compressive strength $f_{\mathrm{NC}}$ and UHPC compressive strength $f_{\mathrm{UHPC}}$. All the collected test data in Section 2 are divided into two parts: 80% for training and 20% for verification.

### 3.2. ANN Uncertainty Quantification

The output results from this ANN model are evaluated for their predictive accuracy with some statistical indicators: *R-square*, mean square error (*MSE*), and covariance (*Cov*) [64]. They are defined as:

(4) $$R-square=1-\frac{\sum_{i=1}^{n}\left(pre_{i}-test_{i}\right)}{\sum_{i=1}^{n}\left(pre_{i}-{\bar{test}}_{i}\right)}$$

(5) $$MSE=\frac{1}{n}\overset{n}{\underset{i=1}{\sum }}{\left(pre_{i}-test_{i}\right)}^{2}$$

(6) $$Cov=\frac{1}{n-1}\overset{n}{\underset{i=1}{\sum }}\left(pre_{i}-{\bar{pre}}_{i}\right)\left(test_{i}-{\bar{test}}_{i}\right)$$

**Figure 6:**
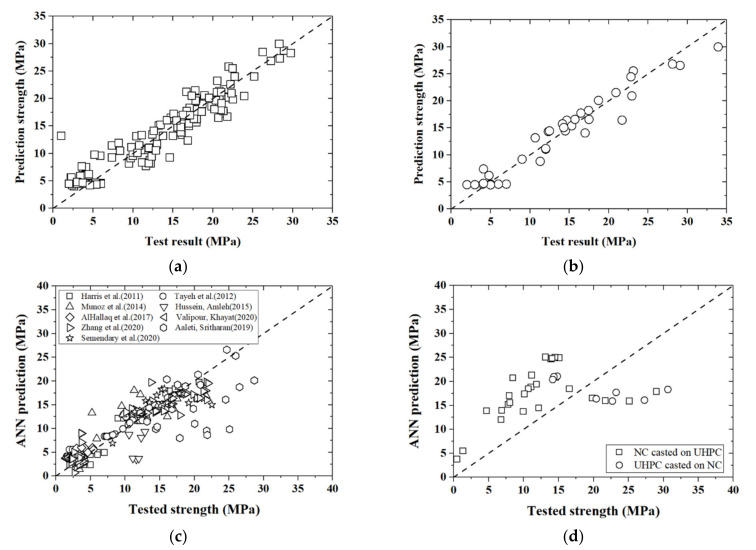
Comparisons between experimental results and ANN predictions. (**a**) Trained group from the database (563 + 338) × 0.8; (**b**) UHPC substrate specimens; (**c**) collected database (563 + 338); (**d**) “improved” slant shear test (35).

It is indicated that lower values of *MSE* and higher values of *R-square* and *Cov* indicate better predictive accuracy; these three indicators were utilized to judge the ANN analyses. As stated above, 80% of the database serves as the training group, while the remaining 20% is treated as the verification group. The indicators *R-square*, *MSE,* and *Cov* were 0.818, 8.06, and 24.56, and 0.794, 8.24, and 26.20 for the training and verification groups, respectively (Table 5, Figure 6a,b). The bond strength predicted from the ANN model was consistent with the existing database, which validates the accuracy of the ANN analyses. The *R-square*, *MSE,* and *Cov* for the overall database (563 + 338 specimens) were 0.82, 8.10, and 34.37, respectively (see Figure 6c and Table 5). Comparisons of the ANN analyses with respect to other existing formulas in the specifications or literature are presented and discussed in Section 4.3.

The ANN model was further applied to our test with 35 specimens for the “improved” slant shear test (see Figure 6d). One innovation of the test was the identification of the casting sequence on bond strength. It is clear that the ANN model overestimated the bond strength of these specimens with NC cast on the existing UHPC, whereas it underestimated the specimens with UHPC cast on the existing NC. The discrepancies between the tested and predictive values for the two groups are presented in Table 5, which indicate a relatively poor predictive quality. Therefore, the casting sequence is a critical influencing factor that cannot be ignored.

### 3.3. Simplified Explicit Model for UHPC–NC Bond Strength

Although the ANN model trained in the previous section yields accurate predictions regarding the bond strength between UHPC and NC layers, it is not convenient for engineering purposes due to the lack of explicit expressions. The implicit calculations in the ANN model are somewhat complicated by the weight vector and bias vectors. To this end, it was necessary to give practical equations relying on the ANN analyses.

To simplify the implicit ANN model, we needed to identify the main influencing factors which affect the bond strength between UHPC and NC materials. With trial-and-error, we deliberatively selected the normal stress level σ, surface roughness R, and compressive strengths of UHPC $f_{\mathrm{UHPC}}$ and NC $f_{\mathrm{NC}}$ materials as the governing factors. Here, the casting sequence was not considered due to the fact that the collected experimental database did not use it as a main factor. However, casting sequence is apparent for the UHPC–NC bond strength according to our test, which is properly treated in Section 5.

According to the collected experimental database, the reference values of these main factors (σ, R, $f_{\mathrm{UHPC}}$ and $f_{\mathrm{NC}}$) are given in Table 6. The maximum, minimum, and average values of these factors were well reflected in the database. To express the influence of the input factors on the UHPC–NC bond strength, a correction function was adopted to explicitly express the ANN analyses as:

(7) $$c\left(\sigma ,R,f_{\mathrm{UHPC}},f_{\mathrm{NC}}\right)=c\left(\sigma \right)\times c\left(R\right)\times c\left(f_{\mathrm{UHPC}}\right)\times c\left(f_{\mathrm{NC}}\right)\;$$

which indicates that each influencing factor would affect the bond strength independently.

**Figure 7:**
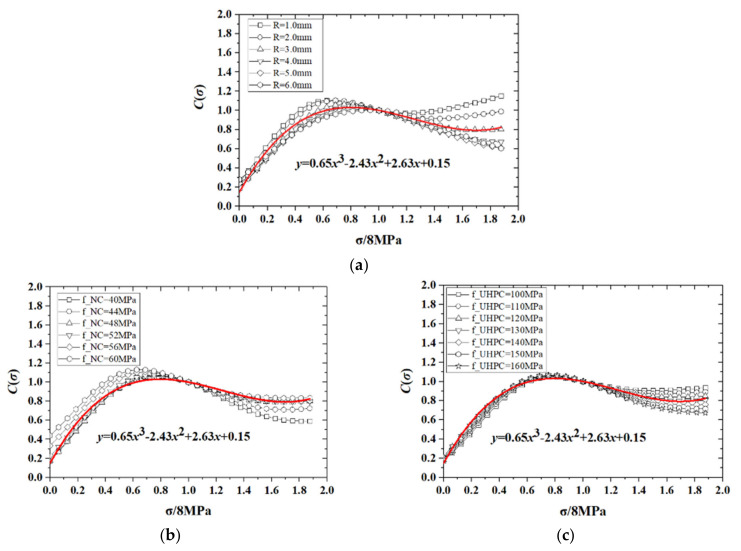
Correction factor $c_{1}\left(\sigma \right)$ with respect to different factors. (**a**) with R; (**b**) with $f_{\mathrm{NC}}$; (**c**) with $f_{\mathrm{UHPC}}$.

**Figure 8:**
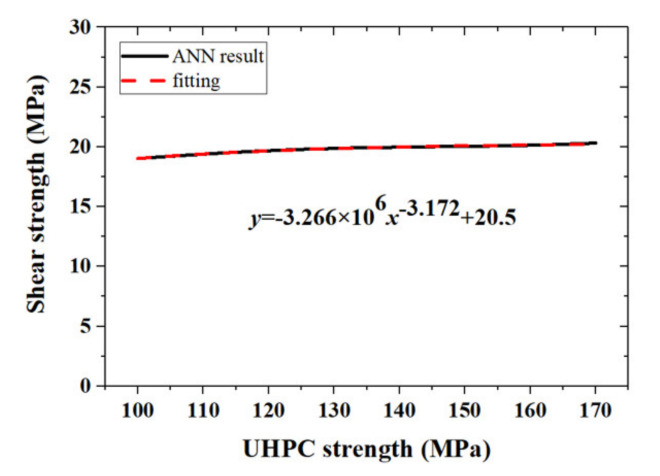
Correction factor $c_{0}\left(f_{\mathrm{UHPC}}\right)$.

**Figure 9:**
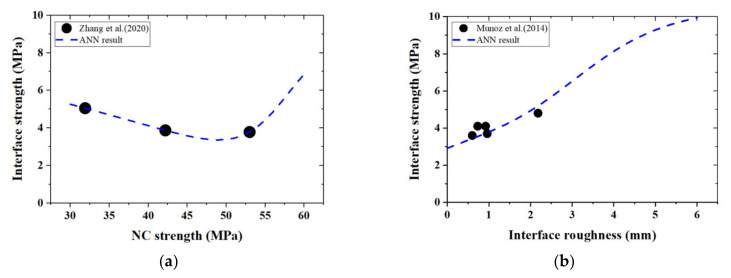
Influence of factors on adhesion. (**a**) Effect of $f_{\mathrm{NC}}$; (**b**) effect of R.

**Figure 10:**
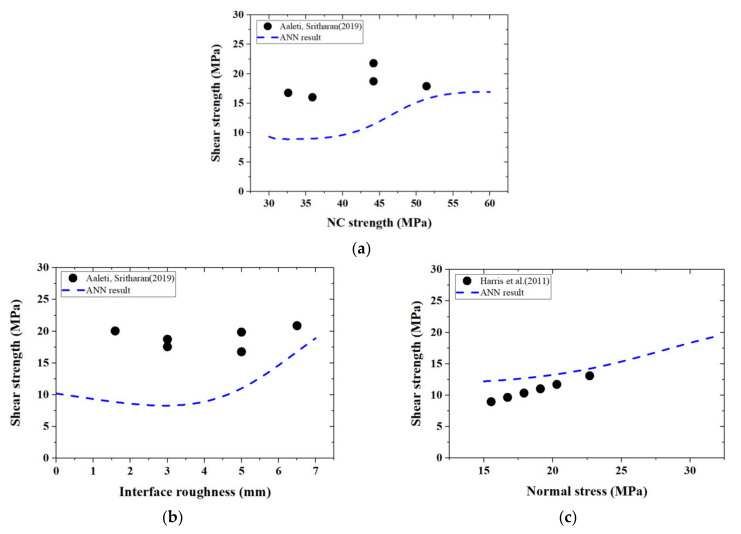
Influence of factors on shear strength. (**a**) Effect of $f_{\mathrm{NC}}$; (**b**) effect of R; (**c**) effect of σ.

The independent functions of the main factors $\left(\sigma ,R,f_{\mathrm{UHPC}}\;\mathrm{and}\;f_{\mathrm{NC}}\right)$ were derived from a step-by-step regression analysis. Taking the function $c_{1}\left(\sigma \right)$ as an example, we first normalized the target value by dividing its average value by σ/8, which is shown as the abscissa in Figure 7. The ANN model was subsequently adopted to calculate the bond strength τ with varying surface roughness R, and compressive strengths of UHPC $f_{\mathrm{UHPC}}$ and NC $f_{\mathrm{NC}}$, which are shown in Figure 7. Finally, the explicit expression of $c_{1}\left(\sigma \right)$ could be obtained through the nonlinear least squares method. Similarly, the explicit expressions for other factors $c_{2}\left(R\right)$, $c_{3}\left(f_{\mathrm{NC}}\right)$, and $c_{4}\left(f_{\mathrm{UHPC}}\right)$ were obtained by this methodology (see Figure 8, Figure 9 and Figure 10). The functions of these main factors are summarized as:

(8) $$c_{1}\left(\sigma \right)=0.65{\left(\frac{\sigma }{8}\right)}^{3}-2.43{\left(\frac{\sigma }{8}\right)}^{2}+2.63\left(\frac{\sigma }{8}\right)+0.15$$

(9) $$c_{2}\left(R\right)=0.39\left(\frac{R}{3}\right)+0.61$$

(10) $$c_{3}\left(f_{\mathrm{NC}}\right)=-10.56{\left(\frac{f_{\mathrm{NC}}}{50}\right)}^{3}+31.13{\left(\frac{f_{\mathrm{NC}}}{50}\right)}^{2}-29.87\left(\frac{f_{\mathrm{NC}}}{50}\right)+12.3$$

(11) $$c_{4}\left(f_{\mathrm{UHPC}}\right)=0.17{\left(\frac{f_{\mathrm{UHPC}}}{130}\right)}^{2}+0.35\left(\frac{f_{\mathrm{UHPC}}}{130}\right)+0.82$$

The correction function in (7) is not sensitive to the compressive strength of UHPC ($f\left(\sigma ,R,f_{\mathrm{NC}},f_{\mathrm{UHPC}}\right)\approx f\left(\sigma ,R,f_{\mathrm{NC}}\right)$), as shown in Figure 9. Therefore, we chose $f_{\mathrm{UHPC}}$ as a reference graph for the study aims and an explicit function $c_{0}\left(f_{\mathrm{UHPC}}\right)$ to describe the relationship between $f_{\mathrm{UHPC}}$ and τ was obtained by fitting the ANN result (Figure 8), as:

(12) $$c_{0}\left(f_{\mathrm{UHPC}}\right)=3.266\times 10^{6}{\left(f_{\mathrm{UHPC}}\right)}^{-3.172}+20.5$$

Finally, we arrived at the explicit expression for the bond strength between UHPC and NC materials based on the ANN results, as:

(13) $$\tau =c_{1}\left(\sigma \right)\times c_{2}\left(R\right)\times c_{3}\left(f_{\mathrm{NC}}\right)\times c_{0}\left(f_{\mathrm{UHPC}}\right)$$

With the above explicit expressions, the implicit ANN model is converted to simplified ones suitable for engineering purposes. The comparisons of the explicit expression with respect to other existing formulas in the specifications or literature are presented and discussed in Section 4.3.

## 4. Improved Empirical Formula Based on Shear-Friction Theory

As stated above, there is an older formula for UHPC–NC bond strength that is still widely accepted by the engineering community. It is recognized that mainstream specifications still concern the interfacial behaviors between normal-strength concrete, rather than UHPC–NC interfaces. Numerous scholars continuously propose formulas for interfacial bond strength between different cementitious materials [65,66]. Nearly all these formulas exclusively rely on the shear-friction theory, which decomposes bond strength into the following two parts: namely, adhesive and frictional forces [32,56]. The conventional shear-friction formulas for UHPC–NC bond strength can be expressed as:

(14) $$\tau =\tau_{c}+µ\sigma$$

where $\tau_{c}$ is the adhesion, σ is the normal stress perpendicular to the interface, and µ is the friction coefficient.

### 4.1. Identification of Influencing Factors

The last section gives the prediction of UHPC–NC bond strength from the ANN analysis (see (5)–(9)). Although it has improved accuracy, it is still not straightforward to employ (5)–(9) for the design aims, as they lack a physical basis and cannot reflect the proper treatment of adhesion and friction forces. To this end, we resort to the conventional shear-friction theory, which is widely adopted in specifications. With the assistance of the ANN analyses, the key influencing factors can be identified from the collected database in Section 2. Relying on this, we were capable of improving the conventional empirical formulas, with (i) solid physical bases compared to explicit expressions from ANN results, and (ii) with greater accuracy compared to the conventional formulas collected from the published literatures.

The trend curve of each design parameter could be obtained by analyzing the collected database in Section 2 with a trained ANN model. As shown in Figure 9 and Figure 10, the interface cohesion and shear strength in the collected database are presented with the ANN prediction curves.

Surface roughness exhibits an apparent influence on both adhesion and friction force (see Figure 9c and Figure 10c) which is consistent with current specifications—AASHTO LRFD [18], Eurocode 2 [17], and CEB-FIP Model Code [16]. The Coulomb friction theory suggests that the increase in surface roughness could improve the mechanical interlock for friction force [49,67]. At the same time, a rougher surface also provides stronger chemical reactions and enhances adhesion [68]. Nevertheless, the relationship between adhesion or shear strength was not linear (see Figure 9c and Figure 10c). The collected slant shear tests suggest that shear strength would increase linearly with the applied normal stress, as shown in Figure 10c, which agrees well with the Coulomb friction theory.

As for the compressive strength of the NC materials, most of the specifications and research papers assume that the interfacial bond strength linearly increases with the NC strength [67,69]. However, the proposed ANN model and the experimental database reject this relation. The splitting test even suggests a decreasing trend in adhesion with respect to NC strength when $f_{\mathrm{NC}}$ is less than 50 MPa, as shown in Figure 9a. It is widely accepted that NC materials with higher strength have lower water–cement ratios [59], which could lead to a weaker interfacial bond strength [61]. Nevertheless, Figure 10a suggests that shear strength increases monotonically with NC strength; this inconsistency may be attributed to the concrete crushing failure that frequently occurs in the conventional slant shear test, which impairs the experimental results and underestimates the interfacial strength of lower NC strength. Moreover, the ANN model also indicates an ignorable effect of UHPC strength on the interfacial bond strength [29], as shown in Figure 9b and Figure 10b. 

In addition, the “*improved*” slant shear test with 35 specimens in Section 2.1 identifies for the first time the effect of casting sequence on interfacial bond strength (see Figure 6b). It suggests that casting UHPC on existing NC materials would lead to a higher bond strength. Zhang et al. [49] obtained the SEM micrographs of the transition zones at the NC–NC and UHPC–NC interfaces, respectively; they pointed out that dense micro-structures would prevent the viscous cement slurry infiltrating into the UHPC component. Comparatively, the micro-structure of the NC materials is porous due to the bleeding bubbles and coarse aggregates, which allow the penetration of UHPC. As a result, casting UHPC on NC is more likely to result in better interfacial bond strength.

### 4.2. Modified Shear-Friction Formula

It is recognized that the shear-friction theory is widely adopted in specifications for interfacial strength between NC materials. It seems indispensable to give a modified formula relying on this theory for UHPC–NC materials that has a solid physical basis. 

To this end, a modified formula is proposed which divides the shear force between UHPC and NC materials into adhesion and friction forces, as:

(15) $$\tau =\underset{\mathrm{Adhension}}{\underbrace{\gamma [a{\left(f_{\mathrm{NC}}\right)}^{2}+bf_{\mathrm{NC}}+c]\alpha R^{\beta }}}+\underset{\mathrm{Friction}}{\underbrace{\gamma \sigma R^{\beta }}}$$

where γ is the coefficient reflection the casting sequence (γ = 0.9 for casting UHPC first and 1.0 for casting NC first), and a = 0.0128, b = −0.7976, c = 18.9780, α = 0.4873, and β = 0.1102 are coefficients determined by the collected database with the least square fitting method. The modified formula considers the effects of casting sequence, surface roughness, normal stress perpendicular to the interface, and compressive strength of NC. The compressive strength of UHPC is not accounted for anymore due to the fact that the ANN results do not show an apparent effect on bond strength.

### 4.3. Comparisons

To verify the accuracy of the developed explicit formula, a comparative study was performed between the implicit ANN model (Section 3.1), explicit formula (Section 3.3), modified shear-friction formula (Section 4.2), and relevant formulas in mainstream specifications and research papers. In this study, AASHTO LRFD [18] were selected for comparison. Moreover, the proposed formulas in Mattock and Hawkins [19], Mattock [70], Mattock [71], and Papanicolaou and Triantafillou [69] were also selected. These formulas are summarized in Table 7.

**Figure 11:**
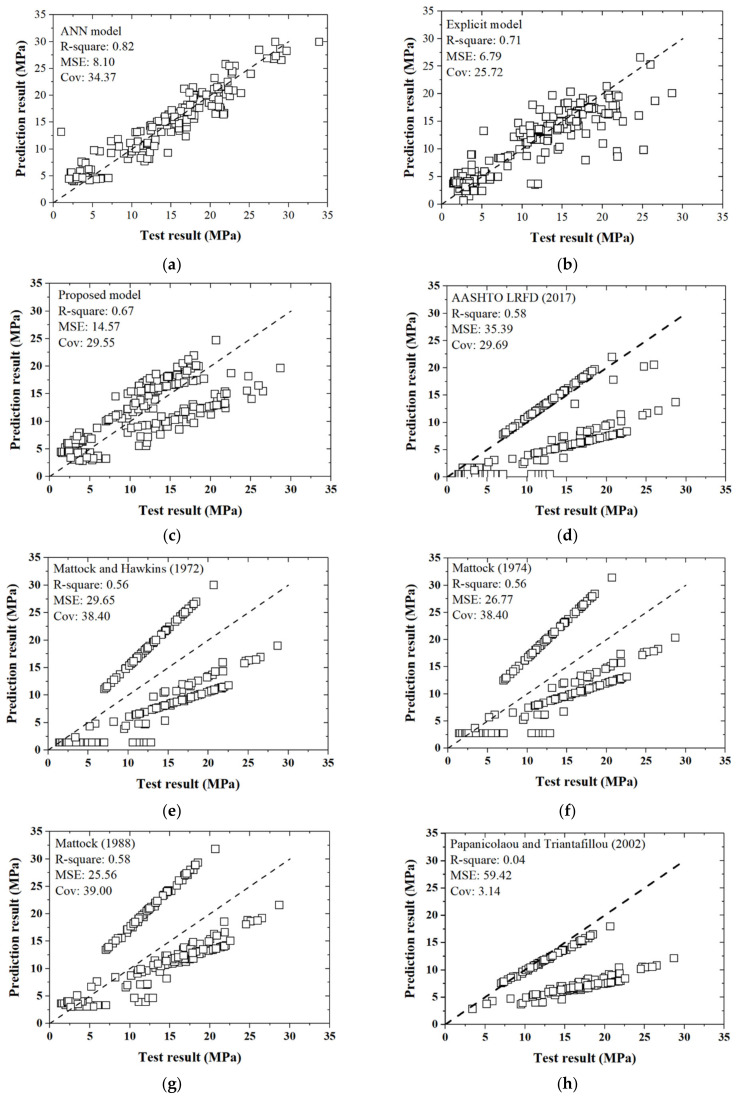
Comparisons of predictive accuracy of different models. (**a**) ANN model; (**b**) explicit formula; (**c**) modified shear-friction formula; (**d**) AASHTO (2017); (**e**) Mattock and Hawkins (1972); (**f**) Mattock (1974); (**g**) Mattock (1988); (**h**) Papanicolaou and Triantafillou (2002).

The relationship between tested results (collected database and our tests) and predictive values from various formulas in Table 7 are presented in Figure 11, and their discrepancies quantified with *R-square*, *MSE*, and *Cov* are compared in Table 8. It can be seen that the implicit ANN model gives the best predictive accuracy with the highest *R-square* of 0.82 and lowest *MSE* of 8.10. The *Cov* of the ANN model is moderate at 34.37. The explicit formula derived from the ANN model in Section 3.3 performs worse than the implicit ANN model, with *R-square* = 0.71, *MSE* = 11.79, and *Cov* = 29.55, respectively. Compared to the ANN model and the explicit formula, the modified shear-friction formula in Section 4.2 yields relatively bigger discrepancies; although, with the predictive accuracy being sacrificed to some extent, the modified shear-friction formula has a solid physical basis and is convenient for engineering purposes. Nevertheless, compared to other formulas in either specifications or research papers, even the modified shear-friction formula shows significant improvements in predictive accuracy.

## 5. Conclusions

This study aimed to give a better prediction of the bond strength between UHPC and NC materials. The conclusions can be tentatively summarized as:

(1) The “improved” slant shear test was devised, and 35 specimens were loaded to failure. It was found that the “improved” test could circumvent concrete crushing and force the occurrence of interfacial debonding, which renders the results more reliable.
(2) It was firstly identified that casting sequence could affect the bond strength between UHPC and NC. Casting NC first, followed by UHPC, could lead to a higher bond strength.
(3) To better predict the bond strength, this study collected 563 specimens in total for the splitting test and 338 specimens for the slant shear test, from published literature in the last few decades. ANN analyses were performed which could give more accurate results, with the following main factors being considered, namely, the normal stress perpendicular to the interface, interface roughness, and compressive strengths of the UHPC and NC materials.
(4) Explicit expressions for the UHPC–NC bond strength were derived based on the proposed ANN model and collected database. Although without a clear underlying physical basis, these explicit expressions are convenient for engineering purposes.
(5) To embrace a physical basis, the conventional shear-friction formula was modified for the UHPC–NC bond strength, which was more straightforward for practical use. The modified formula takes the important factors into account and greatly improves the accuracy of the prediction of UHPC–NC bond strength.

## Figures and Tables

**Table 1:** Specimens for “improved” slant shear test.

Specimen	Quantity	Upper Component	Specimen	Quantity	Upper Component
NU70HS	3	NC	UHPC	70	dashed
NU70HD	3				dotted
NU70S	4				smooth
NU60S	1			60	smooth
NU50S	1			50	smooth
NU70E	9			70	electric pick
NU60E	3			60	electric pick
NU50E	3			50	electric pick
UN70E	2	UHPC	NC	70	electric pick
UN60E	3			60	electric pick
UN50E	3			50	electric pick

**Table 2:** Experimental results of “improved” slant shear test.

Specimen	Quantity	Roughness (mm)	Peak Load (kN)	Normal Stress (MPa)	Shear Strength (MPa)
NU70HS	3	5.00	410.21	5.18	14.03
NU70HD	3	5.00	386.82	4.67	13.23
NU70S	4	1.00	214.31	2.88	7.33
NU60S	1	1.00	26.00	0.72	1.30
NU50S	1	1.00	72.81	3.93	4.68
NU70E	9	3.00	288.87	3.63	9.88
NU60E	3	3.00	422.20	12.06	21.11
NU50E	3	3.00	296.99	16.43	19.09
UN70E	2	3.00	426.29	5.60	14.58
UN60E	3	3.00	427.60	12.13	21.38
UN50E	3	3.00	385.20	22.33	24.76

**Table 3:** Parameters considered in the collected splitting tests.

Literature	Quantity	Design Parameters				
		Roughness	Freeze–Thaw	Age	Material Strength	Moisture
Harris et al. (2011) [51]	47	√	√			
Tayeh et al. (2012) [25]	39	√		√		
Carbonell Muñoz et al. (2014) [22]	284	√	√	√	√	
Hussein, Amleh (2015) [52]	18				√	
Harris et al. (2015) [26]	72	√				
AlHallaq et al. (2017) [53]	18	√			√	
Valipour, Khayat (2020) [54]	60			√	√	
Zhang et al. (2020) [49]	25	√		√		√

**Table 4:** Parameters considered in the collected slant shear tests.

Research	Quantity	Design Parameters				
		Interface Texture	Age	Material Strength	Casting Sequence	Moisture
Harris et al. (2011) [51]	81	√				
Tayeh et al. (2012) [25]	45	√	√			
Carbonell Muñoz et al. (2014) [22]	54	√	√			
Aaleti and Sritharan (2019) [28]	63	√		√	√	
Semendary and Svecova (2020) [60]	15		√			
Zhang et al. (2020) [49]	60	√	√	√		√

**Table 5:** Statistical analysis results.

		*R-Square*	*MSE*	*Cov*
Database (563 + 338)	Training group (80%)	0.818	8.06	34.86
	Verification group (20%)	0.794	8.24	31.52
	Overall	0.82	8.10	34.37
Our test (35)	Casting NC on UHPC	0.22	65.57	15.70
	Casting UHPC on NC	0.49	61.48	8.38

**Table 6:** The main influencing factors.

Factors	Minimum	Maximum	Average
σ (MPa)	0	15	8
R (mm)	0	6	3
$f_{\mathrm{NC}}$ (MPa)	40	60	50
$f_{\mathrm{UHPC}}$ (MPa)	100	170	130

**Table 7:** Relevant formulas in specifications.

Codes	Surface Textures	Expression	Friction Coefficient *µ*	Adhesion *c*
AASHTO (2017) [18]	CIP slab roughened 6mm	$\tau =c+µ\left(A_{v}f_{y}+P\right)/A$	1.0	1.9
	Normal-density concrete monolithically		1.4	2.8
	Low-density concrete roughened 6mm		1.0	1.7
	Normal-density concrete roughened 6mm		1.0	1.7
	Clean concrete, not roughened		0.6	0.52
	Clean concrete reinforced		0.7	0.17
Mattock (1972) [19]	--	$\tau =c+µ\left(\rho f_{y}+\sigma_{n}\right)$	0.8	1.38
Mattock (1974) [70]	--	$\tau =c+µ\left(\rho f_{y}+\sigma_{n}\right)$	0.8	2.76
Mattock (1988) [71]	--	$\tau =cf_{c}^{0.545}+µ\left(\rho f_{y}+\sigma_{n}\right)$	0.8	0.467
Papanicolaou and Triantafillou (2002) [69]	Smooth interfaces	$\tau =c\sqrt{f_{c}}+µ\left(\rho f_{y}+\sigma_{n}\right)$	1.7	0.33
	Rough interfaces		1.4	0.45

**Table 8:** Relevant formulas in specifications.

		*R-Square*	*MSE*	*Cov*
This study	ANN model	0.82	8.10	34.37
	Explicit formula	0.71	11.79	29.55
	Modified shear-friction formula	0.67	14.57	25.72
Specifications	AASHTO (2017)	0.58	35.39	29.69
Research papers	Mattock and Hawkins (1972)	0.56	29.56	38.40
	Mattock (1974)	0.56	26.77	38.40
	Mattock (1988)	0.58	25.56	39.00
	Papanicolaou and Triantafillou (2002)	0.04	59.42	3.14

## Data Availability

All data, models, or code generated or used during the study are available from the corresponding author by request.
